# Molecular characterization of some isolates of rabbit viral hemorrhagic disease (VHD) in Egypt from 2014 to 2019

**DOI:** 10.5455/javar.2021.h528

**Published:** 2021-09-19

**Authors:** Hanaa Awad El-Samadony, Hoda Mohammed Mekky, Aly Mohammed Ghetas, Aalaa Samir Saad

**Affiliations:** 1 Poultry Diseases Department, Animal Health Research Institute, ARC, Giza, Egypt; 2 Poultry Diseases Department, Veterinary Research Division, National Research Centre, Giza, Egypt; 3 Food Hygiene Department, Animal Health Research Institute, ARC, Giza, Egypt

**Keywords:** Rabbit viral hemorrhagic disease, genetic identification, phylogenetic analysis, VP60, Egypt

## Abstract

**Objective:**

Rabbit viral hemorrhagic disease (VHD) is a transmittable and lethal viral illness of rabbits. In this study, genetic identification and genetic analysis of the rabbit hemorrhagic disease virus (RHDV) was made in three governorates in Egypt from 2014 to 2019.

**Materials and Methods:**

Livers from 18 freshly dead rabbits, which was guessed to be VHD epidemics in Egypt (Giza, Menofia, and Fayoum governorates) from 2014 to 2019, were examined for RHDV. The examination was based on the hemagglutination assay (HA) test against different mammalian (human O-type and sheep) and avian (chicken and pigeon) erythrocytes, reverse transcriptase-polymerase chain reaction (RT-PCR), and sequencing of the segment of VP60.

**Results:**

33% of the examined samples’ virus titers were 5 log_2_ to 8 log_2_ hemagglutination of human O-type erythrocytes when compared to 28%, 11%, and 28% of sheep, chicken, and pigeon erythrocytes, respectively. Four RHDV isolates out of eight RT-PCR positives were sequenced and phylogenetically analyzed. Sequenced isolates were designed and submitted to GenBank with accession numbers MN904506, MN904507, MN904508, and MN904509. These four RHDV isolates were related to classic G3 (GI.1d/RHDV). Twelve amino acid differences were detected between the vaccine strain sequence (Giza-2006) and RHDV isolates. Amino acid differences at 416, 423, and 476 positions seem interesting as they changed polarity that could change the protein structure and affect host interaction.

**Conclusions:**

There is antigenic variation between circulating RHVD strains and the vaccinal strain. This may be the leading cause of vaccination failure and may increase the need to check out the vaccination program against RHVD.

## Introduction

The rabbit industry is promising when it comes to increasing Egyptian revenue, which can help cover meat shortage. Egypt is considered among the top five rabbit meat producers worldwide. Small to medium-sized rabbit producers contribute to a high production rate [1,2]. Rabbit hemorrhagic disease (RHD) extends worldwide and is accompanied by high fatality rates [3].

Rabbit hemorrhagic disease virus (RHDV) is included in the genus *Lagovirus* of the family Caliciviridae [4]. RHDV is an RNA virus (single-stranded) non-enveloped, with about 7,437 nucleotides. The genomic RNA consists of two overlappings (open reading frames, ORFs). ORF1 (7,034 nucleotides) encodes a huge multiprotein. ORF1 is split into seven non-structural viral proteins and a structural VP60. The shorter ORF2 (353 nucleotides) encodes viral protein VP10 [5]. The subgenomic RNA (2.2 kb) encodes VP60 and VP10. The VP60 contains the specific antigenic epitope, which shows the host immunological reaction target versus RHDV [6]. Thus, molecular studies were carried out on partial and complete sequences of the *VP60* gene to detect the genetic variations between RHDV strains [7–9].

RHVD-affected animals in 12–36 h develop a fever followed by sudden death of its onset in peracute type. Animals with acute RHD survive longer, but suffer from general illness such as depression, off food, redness of the ocular mucous membrane, and in several situations from pulmonary dysfunction (e.g., dyspnea, cyanosis, and blood-stained frothy rhinal secretion), ocular or rhinal hemorrhages. Some rabbits also have disorders of nerves as restlessness, backward arching of the head and neck, and paddling [10]. The subacute type has temperate signs, and almost all rabbits live and produce protective antibodies versus RHDV reinfection. The chronic form is characterized by jaundice, which is severe, generalized, off food, and lethargy. Rabbits resort to die after 1–2 weeks, but those who overcome the disease have a solid seroconversion [3]. 

RHDV strains are classified into three subtypes: RHDV classic (G1–G5), RHDVa variant, and RHDVb variant [11]. Lately, thay have been categorized into two genotypes: GI.1 that has different forms, GI.1a (G6/RHDVa), GI.1b (G1), GI.1c (G2), and GI.1d (G3–G5), and GI.2 [12]. These forms show antigenic, genetic, and pathogenicity differences, affecting virus adaptation and spread [8,9]. RHD outbreaks caused by GI.1a are characterized by a 70%–90% mortality rate in rabbits older than 4–6 weeks. But younger rabbits are less susceptible. Clinical signs and mortality rate (5%–70%) were determined in adults and even in rabbit kittens from 15 to 20 days old infected with GI.2 [10]. These variants also have hemagglutinating, non-hemagglutinating, or variable hemagglutinating activity [13].

The immunogenic protein and the main viral structure of RHDV are VP60 [14]. It comprises three parts: the N-terminal arm, S, and a short hinge, and P splits into two portions P1 and P2. The VP60 protein variance caused differences in epidemiological diversity, molecular, immunizing agent, and body defenses between three RHDV subtypes. [15,16].

In Egypt, the first reported HVD outbreak was in 1991 [17]. Subsequently, the disease outbreaks were reported in different governorates [18,19]. Despite several vaccination programs, different RHDV variants belonging to GI.1 were identified in vaccinated [20,21] and non-vaccinated rabbits [22]. Recently, RHDV strains relating to GI.2 (RHDV2/b) have been identified from outbreaks in vaccinated rabbit flocks [23]. For effective control of RHDV outbreaks, continuous monitoring of RHDV circulating in Egypt is in demand. Thus, the study goal was to molecular characterize RHDV from 18 suspected RHDV-infected farms in three different governorates in Egypt (Giza, Menofia, and Fayoum) from 2014 to 2019.

## Materials and Methods

### Ethical approval

The study was carrid out according to 10 principles of the Declaration of Helsinki [24]. All examined tissues were disposed of according to biosafety procedures under the committee’s supervision.

### Sample collection

Livers from freshly dead rabbits [approximately 5%–10% livers from each farm representing one sample (clinical case) which were delivered to our lab for examination] were collected from 18 suspected RHDV cases [3 unvaccinated (20 examined livers) and 15 vaccinated (100 examined livers)] in 3 different governorates in Egypt (Giza, Menofia, and Fayoum) during the period from 2014 to 2019, with average age of 1–3 months and from various breeds (New Zealand, Albino, and Baladi) with the history of vaccination (killed vaccine). Suspected rabbits were those suffering from lacrimation, ocular hemorrhages, depression, off food, reddish conjunctiva, blood-stained frothy nasal discharge, and fragile liver with focal lobular necrosis, accompanied by an enlarged congested hemorrhagic spleen and bloody kidneys, as well as hemorrhagic tracheitis, congested edematous, and hemorrhagic lungs. The collected samples were kept at −80°C for diagnosis.

### Sample preparation

10% suspension of liver homogenate in phosphate buffered saline (PBS) at pH 7.2–7.4 was prepared, filtered through a cheesecloth, and centrifuged at 5,000× for 15 min [25]. The supernatant was stored at −80°C.

### Erythrocyte suspension

Four parts of freshly collected blood samples from human O-type, chicken, sheep, and pigeon were added to one part 4% sodium citrate (as an anticoagulant). For washing the erythrocyte, the volume of PBS (pH 7.1) equal to the volume of erythrocytes was added and then the suspension was centrifuged as 500× for 5 min. The supernatant was decanted. The washing step and decanting were repeated. Then, 0.5% erythrocyte suspension was prepared (0.5 ml packed cells to 100 ml PBS at pH 7.0–7.2) [26].

### Hemagglutination assay (HA) test

The HA test was carried out to identify RHVD according to [27] in 96 V-shaped wells microtitration plate. The HA titer that correlated to the highest dilution, producing complete agglutination of erythrocytes, was taken.

### Reverse transcriptase-polymerase chain reaction

Extraction of RHVD mRNA was carried out using the PathoGene-spin™DNA/RNA Extraction Kit following the manufacturer’s instructions. The used primers were P33: 5′-CCA CCA CCA ACA CTT CAG GT-3′ and P34: 5′-CAG GTT GAA CAC GAG TGT GC-3′ targeting 538 best pair (bp) [28]. The reactions were performed in one-step reverse transcriptase-poly­merase chain reaction (RT-PCR) (System Biolab Company), that were warmed up at 50°C for 15 min and then at 95°C for 15 min; then the 35 cycles of 95°C for 1 min, 56°C for 1 min, 72°C for 2 min, and finally 72°C for 10 min [28].

### Partial sequencing of VP60 gene

The PCR product sequencing was carried out by Sigma Company using the Sanger sequencer in two directions. Sequences were analyzed using BioEdit program version 7.1.5 [29]. Bioedit software was used for comparison between the obtained sequences and the others carried out in the GenBank. The neighbor-joining phylogenetic analysis was carried out by MEGA program version 7 [30].

### Analysis of the sequenced part of VP60 gene

RasWin Molecular Graphics program version 2.7.5.2. was used to predict the three-dimensional structure of the VP60 P domain (PDB ID: 4X1W) to analyze the biological significance of functional divergence-related sites. The N-linked glycosylation sites of VP60 were predicted using http://www.cbs.dtu.dk/services/NetNGlyc/.

## Results

### HA test

HA was carried out on the liver homogenates from the suspended HVD rabbits against different mammalian (human O-type and sheep) and avian (chicken and pigeon) samples. In this test, agglutination of dilution more than 1/16 is considered positive [24]. As shown in Table 1, a high percentage of positive samples (33%) had 5 log_2_ to 8 log_2_ HA titer by human O-type erythrocytes compared to 28%, 11%, and 28% sheep, chicken, and pigeon erythrocytes, respectively.

### Reverse transcriptase-polymerase chain reaction

In this study, applying one-step reverse transcriptase-polymerase chain reaction (RT-PCR) on liver samples using specific primers targeting the VP60 gene of RHVD revealed the presence of the amplified band at the expected size 538 bp in 8 (44%) samples out of the 18 examined samples. The positive cases were five from vaccinated farms and three from unvaccinated farms.

### Sequencing of the amplified part of the VP60 gene

Sequenced isolates were designed and submitted to the GenBank with accession numbers MN904506, MN904507, MN904508, and MN904509.

### Phylogenetic analysis

**Figure 1.**
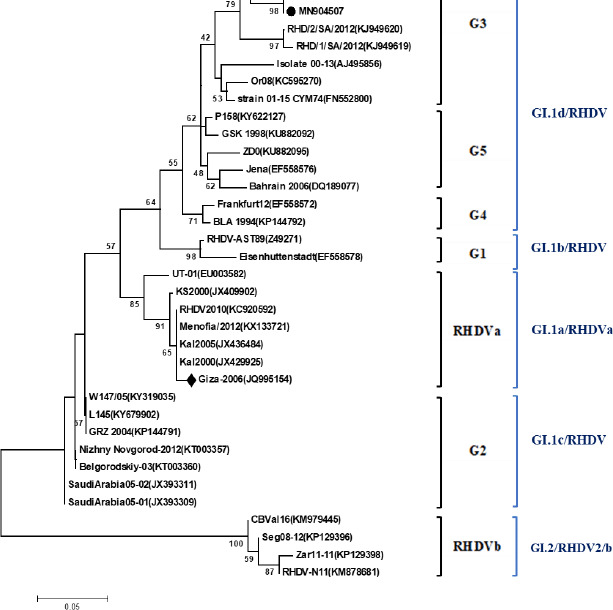
Phylogenic tree established on sequences and segments of the RHDV *VP60* gene. • refers to viruses isolated in the current study and ♦ is the Giza-2006 vaccinal strain.

**Figure 2.**
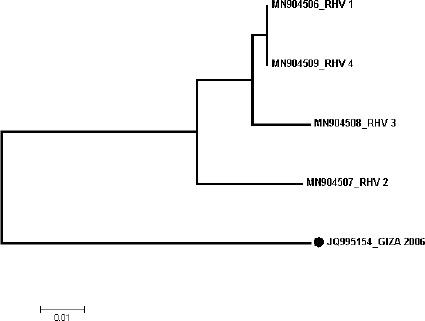
Phylogenic tree established on amino acid sequences of the RHDV *VP60* gene between viruses isolated in the current research and the vaccinal strain. The black dot refers to the vaccinal strain.

Four RHDV strains out of eight RT-PCRs were positive (selected randomly) sequenced. The phylogenetic tree containing the 4 identified strains and 33 reference strains was constructed based on a partial sequence of VP60. As shown in Figure 1, the four strains identified in this study are related to GI.1d/RHDV. Yet, the used RHDV vaccine in Egypt, Giza-2006 (JQ995154), belongs to GI.1a/RHDVa. Furthermore, we reported amino acid differences between RHDV strains identified in this study and the vaccinal strain (Giza-2006) based on partial VP60 sequence. We found 12 amino acid differences (Table 2): 7 differences between Giza-2006 and the 4 strains (RHV-1, RHV-2, RHV-3, and RHV-4) identified in this study (I 412 G, T 416 A, P 423 S, V 434 I, A 476 S, S 480 T, L534 F), 2 differences between Giza-2006 and MN904507 (RHV-2) strain (N 414 S, V 467 I), 2 differences between Giza-2006 and MN904508 (RHV-3) strain (T 449 S, P 450 A), and 1 difference between Giza-2006 and both MN904506 (RHV-1) and Mn904509 (RHV-4) strains (N 481 S). The phylogenic tree of protein (Fig. 2), Giza-2006, is in a different cluster other than the four strains of RHDV under analysis.

**Table 1.** HA titers log_2_ of suspected RHVD suspected samples against different mammalian and avian’s erythrocytes.

	Number of samples/(HA titer to human O type log_2_)	Number of samples/(HA titer to sheep RBCs log_2_)	Number of samples/(HA titer to chicken RBCs log_2_)	Number of samples/(HA titer to pigeon RBCs log_2_)
	1/0	2/0	5/0	2/0
	2/1	2/1	2/1	0/1
	2/2	4/2	3/2	4/2
	4/3	3/3	3/3	5/3
	3/4	2/4	3/4	2/4
	**3/5**	**4/5**	**2/5**	**1/5**
	**1/6**	**1/6**	**0/6**	**3/6**
	**2/8**	**0/8**	**0/8**	**1/8**
**Total positive**	**(6/18) 33%** ^a^	**(5/18) 28%** ^a^	(2/18) 11%*	**(5/18) 28%** ^a^

Bold numbers indicate that HA is positive and that the HA titers are greater than 4 log_2_.

a HA total positive percentage (No. of samples with HA titers more than 4 log_2_/total number of samples).

### Amino acids differences

**Figure 3.**
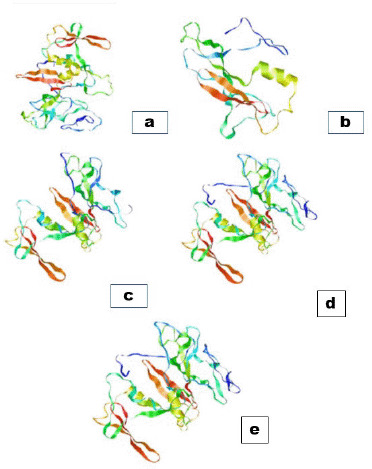
Three-dimensional structure of VP60 P domain. Ribbon representation of the VP60 protein structure. (a) (MN904506). (b) (MN904507). (c) (MN904508). (d) (MN904509). (e) Giza-2006 vaccinal strain.

The three-dimensional structure of VP60 (Fig. 3) and N-linked glycosylation sites of VP60 showed that the MN904506 strain had an N-linked glycosylated site at positions 70 aa and 98 aa. However, MN904507 strain contained N-linked glycosylated sites at 63 aa, 70 aa, and 91 aa. MN904508 strain sites 3 aa, 84 aa, 91 aa, and 112 aa were predicted as N-linked glycosylated sites. However, N-linked glycosylated sites of the MN904509 strain were 3 aa, 84 aa, and 112 aa, while the vaccine strain JQ995154 N-glycosylated predicted sites were 3 aa, 24 aa, 91 aa, and 112 aa.

## Discussion

In this study, genetic characterization of RHDV from 18 suspected RHDV cases in 3 different governorates in Egypt from 2014 to 2019 was carried out.

HA testing of suspected samples against different mammalian and avian erythrocytes is shown in Table 1. OIE [25] recommended HA test using human type-O erythrocytes [25], but it is not easy to obtain and keep this type of erythrocytes [31]. Usage of different avian erythrocytes were used to test how accurate HA results are compared to that obtained by using human O-type erythrocytes. High HA-positive percent and titer were found with human O-type erythrocytes, followed by sheep and pigeon erythrocytes. The result agreed with Sahar’s [32] study that human O-type erythrocytes in the HA test had high HA titer, followed by sheep and pigeon erythrocytes. Although the HA test is a preliminary diagnostic test for RHDV [25], it is not accurate since RHDV strains have non-hemagglutinating or variable hemagglutinating activities, which are reported [13].

**Table 2.** Amino acid differences between the four RHDV strains that were identified and the Giza-2006 vaccinal strain used in Egypt based on a partial VP60 sequence.

AA positions	Giza-2006	MN904506	MN904507	MN904508	MN904509
412	I	G	G	G	G
414	N	N	S	N	N
416	T	A	A	A	A
423	P	S	S	S	S
434	V	I	I	I	I
449	T	T	T	S	T
450	P	P	P	A	P
467	V	V	I	V	V
476	A	S	S	S	S
480	S	T	T	T	T
481	N	S	N	N	S
534	L	F	F	F	F

A = alanine, F = phenylalanine, G = glycine, I = isoleucine, L = leucine, N = asparagine, P = proline, S = serine, T = threonine, V = valine.

Prevention of HVD outbreaks depends on the vaccination of rabbits by an effective vaccine closely related to the circulating RHDV strains. In Egypt, an inactivated vaccine based on classic strain has been developed [33]. After the emergence of variant strain in 2008, the classic strain has been replaced [34]. This variant strain was characterized as an RHDVa variant based on sequence analysis of the *VP60* gene’s full length [35]. The four RHDV strains identified in this study belonged to GI.1d/RHD (Fig. 1). Vaccine strain (Giza-2006) used for vaccination of those rabbit flocks under investigation belonged to GI.1a/RHDVa. Also, RHDV stains related to GI.1 and GI.2 were identified in vaccinated and non-vaccinated rabbits [21].

In our study, partial sequences of the *VP60* gene’s nucleotide of four RHDV strains (randomly selected) were analyzed with the Egyptian vaccinal strain (Giza-2006) to detect any potential genomic differences. The four RHDV strains belonged to GI.1d/RHD compared to the most commercially used vaccine in Egypt, Giza-2006, which belonged to GI.1a/RHDVa. Furthermore, amino acid differences between RHDV strains identified in this study and vaccinal strain (Giza-2006) were reported based on a partial *VP60* gene sequence. We found 12 amino acid differences (Table 2) that could change the shape of the VP60 protein (Fig. 2). The difference in amino acids positions 416, 423, and 476 are attractive because these differences change amino acid polarity that could affect VP60 protein folding at these positions [36]. That leads us to deduce that the Giza-2006 vaccine showed a different antigenic index and surface probability profiles from other local circulating variant RHDV strains that may be responsible for emerging new outbreaks despite vaccination*.* Amino acid position 476 has been described as one of the positively selected codons and potential sites of N-glycosylation, which is principal for protein structure, after analysis of 43 RHDV VP60 complete and pathogenic sequences worldwide distributed and available at GenBank.

N-glycosylation and phosphorylation were mentioned in that they had a principal function in viral infection, different biological functions of proteins, replication, and translation [37,38].

N-glycosylation and phosphorylation mentioned a significant function in viral infection, different biological functions of proteins, replication, and translation [37,38). Analyses of the biological significance of the functional N-glycosylation sites of different subtypes of RHDV (Fig. 2) revealed some potential active N-glycosylation sites located in the active domain on the outer surface of RHDV VP60.

## Conclusion

There is antigenic variation between the circulating RHVD strains and the vaccinal strain that may be a potent reason for vaccination failure. Thus, continuous monitoring of the existing RHDV strains circulating in Egypt is lso required. A vaccination challenge study is needed to detect how commercially available vaccines can protect rabbits against RHDV strains. Further regular researches are necessary to establish effective vaccination strategies to prevent RHVD outbreaks. RHVD outbreaks overcome builds on the vaccination of rabbits by effective vaccines closely related to the circulating RHVD strains. Also, there is a requirement to analyze the VP60 protein by other molecular methods such as Western blot immunochemistry.

## List of abbreviations

VHD: viral hemorrhagic disease; VP60: capsid protein viral protein; RHDV: rabbit hemorrhagic disease virus; ORFs: open reading frames; NTA: N-terminal arm; S: shell; P: protrusion; PBS: phosphate buffered saline; h: hour; RT-PCR: reverse transcriptase-polymerase chain reaction; bp: base pair.
